# An Unusual Complication in Plastic Periodontal Surgery

**DOI:** 10.1155/2020/8824246

**Published:** 2020-11-28

**Authors:** Jorge Vagarinho, Sara Sardinha, Ricardo Alves

**Affiliations:** ^1^Instituto Universitário Egas Moniz (IUEM), Portugal; ^2^Centro de Investigação Interdisciplinar Egas Moniz, Portugal

## Abstract

**Introduction:**

Oroantral communications may arise as a result of pathological processes or iatrogenic situations, particularly in oral surgery and implantology, but are uncommon in periodontal plastic surgery. *Case Presentation*. A healthy 37-year-old female patient with 5 mm Miller Class II recession on the maxillary left second molar was referred for a root coverage procedure. While preparing the recipient bed for the graft, an oroantral communication was created. Schneiderian membrane was sutured to reduce the dimension of the communication and covered with a connective tissue graft. Finally, the flap was laterally and coronally moved, and the patient instructed about postoperative precautions. This procedure allowed to achieve a complete closure of the oroantral communication and good root coverage after an 8-month follow-up.

**Conclusions:**

Different authors described different techniques that can be used to close oroantral communications. Nevertheless, in this clinical case, it was shown that the oroantral communication may be closed without having to postpone the periodontal plastic surgery.

## 1. Introduction

Gingival recession consists in an apical migration of the gingival margin with exposure of the root surface [1]. In order to maintain a healthy dentogingival junction, a certain amount of keratinized tissue is needed [2]. There are several techniques described for gingival recession treatment, but connective tissue graft is considered the gold standard [3]. A successful procedure may not only lead to an esthetic improvement, but also to a functional correction [4].

Usually the connective tissue grafts and free gingival grafts are frequently harvested from the palate. There are some techniques described in literature for harvesting a connective tissue graft with the purpose to achieve primary intention palatal wound healing, the trap-door approach, and the single incision technique [5].

The most frequent complications in periodontal plastic surgery are pain, inflammation, bleeding, flap necrosis, and infection [3].

There are some known factors associated with postoperative complications like duration of the intervention, surgical extension, smoking, surgeon expertise, and nature of the surgical intervention [6].

An oral antral communication defines itself as a pathological continuity between the oral cavity and the maxillary sinus [7, 8]. Different surgical techniques have been described for closing oroantral communications, such as the palatal rotation flap, the trapezoidal flap, and the Bichat's fat pad graft [7]. This pathology has a variety of etiological factors, some of those causes are traumatisms, tumors, cysts, iatrogenesis [9], implant surgery [10], and commonly, after extraction of maxillary molars [11], but are unusual in periodontal plastic surgery. The oroantral communication diagnosis can be made by the Valsalva maneuver: the patient is instructed to expel air against closed nostrils, while the clinician checks if air hisses from the fistula into the mouth [10].

If an oroantral communication is confirmed, primary closure is important to avoid contamination that could lead to impaired healing and chronic sinusitis [8].

## 2. Case Presentation

**Figure 1:**
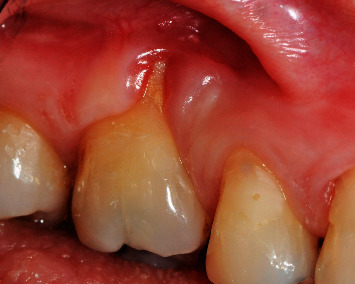
Gingival recession associated to the left second upper molar showing a mucogingival defect.

**Figure 2:**
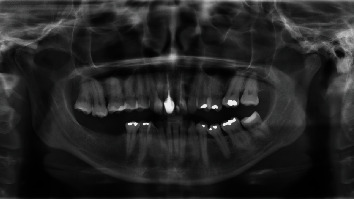
Patient's orthopantomography showing maxillary sinus pneumatization and absence of first left upper molar.

A 37-year-old female Caucasian patient was referred to the periodontology department for evaluation of a gingival recession on the maxillary left second molar. The patient's medical history revealed no systemic diseases or allergies. The clinical (Figure 1) and radiographic (Figure 2) evaluation revealed 5 mm Miller Class II recession [12] on the second molar buccal aspect. There was discomfort during tooth brushing, described by the patient, and permanent inflammation in this area. The probing depths were ≤2 mm, and apically to the recession, there was absence of keratinized gingiva. The proposed treatment was a connective tissue graft combined with a laterally displaced flap.

**Figure 3:**
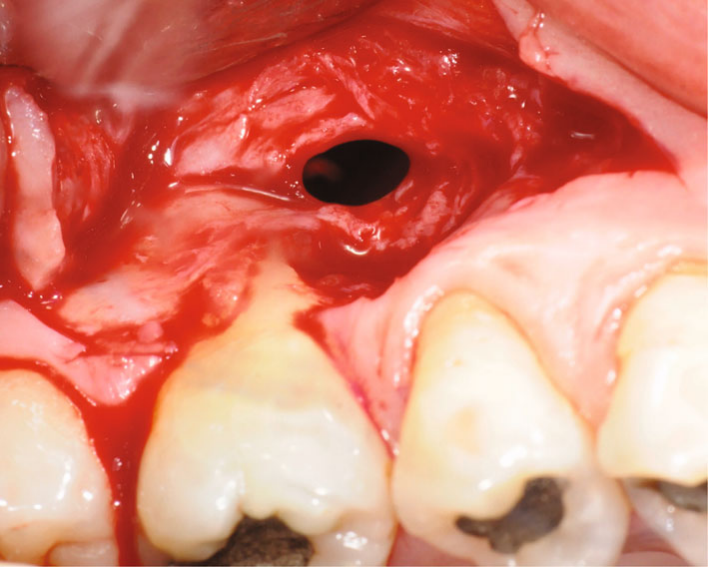
Oroantral communication after flap dissection nearby the MV root of tooth no. 17.

Prior to the surgery, the patient underwent one session of scaling and polishing and received oral hygiene instructions in order to reduce the local inflammation. After explaining the objectives of the surgery, a written informed consent was obtained. The surgery started with the administration of local anesthesia and careful scaling of the exposed root surface, and a partial-thickness flap dissection was extended down into the vestibule, permitting passive coronal positioning of the flap. While preparing the flap, an oroantral communication was found apically to the first molar mesial buccal root with 3 mm diameter (Figure 3).

**Figure 4:**
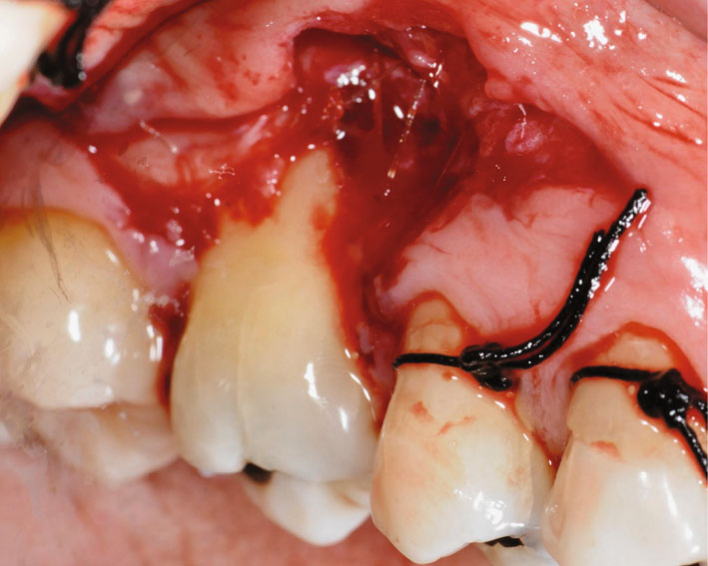
Suture of Schneider membrane with absorbable 6/0 polyglycolic acid suture.

**Figure 5:**
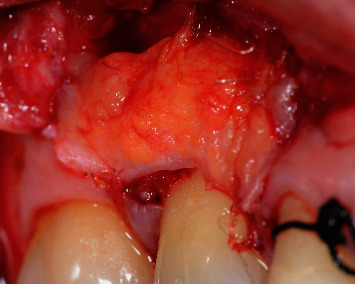
Connective tissue graft adaptation to the recipient bed.

**Figure 6:**
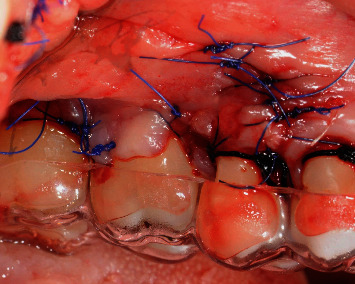
Flap laterally and coronally displaced sutured with 5 0' nylon suture.

Oroantral communication closure started by suturing the Schneider membrane with a 6 0' absorbable suture (monofilament polyglecaprone suture, Surgiclryl-Monofast ®SMI-Belgium) to close the perforation (Figure 4). A connective tissue graft was harvested from the palate according to the technique described by [13]. The recipient bed was measured as well as the palate thickness. Using a #15 blade, oriented perpendicular to the palatal surface, a single incision was made 3 mm apical to the gingival margin of the maxillary teeth. The partial-thickness dissection was made as apically as needed to obtain a graft measuring 12 mm length and 7 mm width. The connective tissue was elevated from the palate, and the donor site was sutured with 4/0 silk sutures (Silk UPS braided, SMI-Belgium) applying pressure against the palate. The connective tissue graft was sutured to the recipient bed with 6 0' absorbable suture (monofilament polyglecaprone suture Surgiclryl-Monofast ®SMI-Belgium) (Figure 5), and the flap laterally and coronally moved and sutured with 5 0' nylon suture (monofilament SERALON, SERAG Wiessner) (Figure 6).

**Figure 7:**
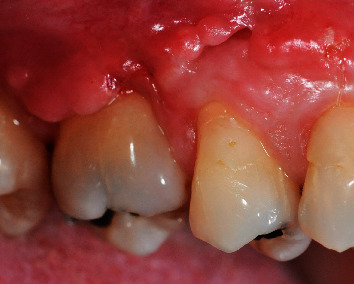
Two weeks postop healing. Apically to the second premolar, there is an incomplete closure due to a noncompliant patient.

**Figure 8:**
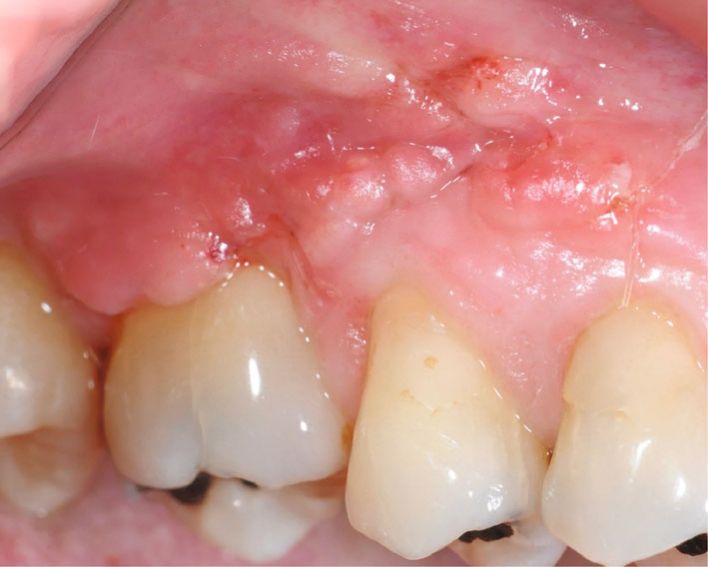
Four weeks postop healing with complete wound closure.

A surgical stent was given to the patient to use during the following week (Figure 6). The patient was instructed to the following: apply ice, in order to reduce the swelling; avoid suction and not to blow her nose; have a soft diet and not to brush the area. She was also recommended to refrain from physical exercise for a week. Chlorhexidine 0.12%, antibiotic, and anti-inflammatory drugs were prescribed (Figures 7 and 8).

## 3. Clinical Outcomes

**Figure 9:**
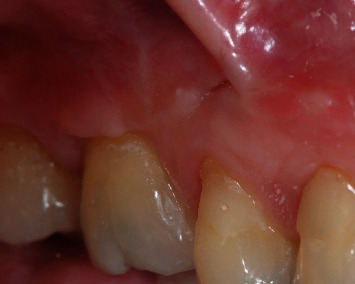
Two months of healing showing no signs of flap diescency.

**Figure 10:**
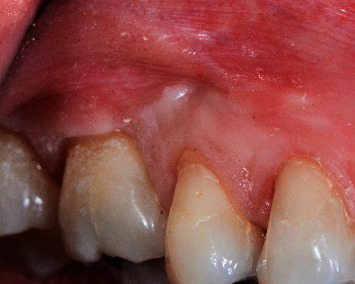
Eight months postop healing. Complete communication closure and complete root coverage with no signs of gingival inflammation—after.

The surgery led to an almost complete root coverage, augmented width of keratinized mucosa, and probing depths < 2 mm with no bleeding on probing (Figures 9 and 10). Patient reports significant reduction in tooth hypersensitivity and absence of discomfort when brushing.

## 4. Discussion

Periodontal plastic surgery procedures are used to improve the esthetic conditions and other clinic aspects, such as the clinical attachment level and width of keratinized tissue, by coverage of previously exposed root surfaces [14].

Commonly, oroantral communication may happen due to pathologic or iatrogenic causes. Different techniques have been proposed to achieve a complete oroantral communication closure after oral surgery procedures, but in periodontal plastic surgery, this is an infrequent event. Several flap designs are used to promote the coverage of these defects, such as buccal and palatal soft tissue flap techniques and their modifications [15].

In the presented case, closure was made in three layers: first, by suturing the Schneider membrane with 6/0 absorbable suture. Secondly, the area was covered with a connective tissue graft harvested from the anterior palate and ultimately a coronally and laterally displaced flap.

The patient was medicated with antibiotics (amoxicillin 1 g bid, 8 days); in the event of an allergy to penicillin, the amoxicillin could be substituted by claritromicillin 500 mg, bid, for 8 days; NSAIDS (ibuprofen 600 mg bid, 4 days); and nasal decongestant (pseudoephedrine 30 mg bid, 3 days), in order to prevent sinus infection and pain and reduce inflammation.

After two weeks postop, there was still a small communication with the sinus and an incomplete wound healing (Figure 7). While interviewing the patient, she assumed to have blown her nose on several occasions. Gentle debridement, irrigation with saline was made together with postop instruction reinforcement. After 4 weeks postop, complete closure of the oroantral communication was achieved (Figure 8). The patient reported that when her first left molar was extracted, some years ago, an oroantral communication occurred. After the loss of a maxillary tooth and reduction of masticatory forces, the sinus wall tends to become thinner by pneumatization of the maxillary sinus. The sinus pneumatization and the previous OAC justified the occurrence of this rare complication. Due to traumatic causes like tooth extraction, when the alveolar bone is totally absent in some places, the sinus mucosa may be in immediate contact with the oral mucosa. In this condition, the Schneiderian membrane cannot usually be kept untouched [16].

To the best of our knowledge, there is no similar case in literature describing an oroantral communication during a periodontal plastic surgery procedure. This case also showed that the periodontal procedure may be completed without compromising the end result (Table 1).

## Figures and Tables

**Table 1:** Summary.

Why is this case new information?	Even though the oroantral communication is considered a common surgical complication, it is not usually described after periodontal plastic surgery procedures.
What are the keys to successful management of this case?	To be successful in a case like this is important to have tissue stability after suturing and a good adherence to the postoperative care instructions.
What are the primary limitations to success in this case?	Patient compliance with postoperative instructions can limit the results of this technique.

## Additional Points

*Summary Sentence/Key Findings*. The creation of an oroantral communication during recipient bed preparation can be adequately managed without affecting the final outcome of root coverage procedures.

## References

[1] Wennström J. L. (1996). Mucogingival therapy. *Annals of Periodontology*.

[2] Edel A. (1974). Clinical evaluation of free connective tissue grafts used to increase the width of keratinised gingiva. *Journal of Clinical Periodontology*.

[3] Aguirre-Zorzano L. A., Fuente A. M. G. D., La E.-F. R., Marichalar-Mendía X. (2017). Complications of harvesting a connective tissue graft from the palate. A retrospective study and description of a new technique. *Journal of Clinical and Experimental Dentistry*.

[4] Chambrone L., Pannuti C. M., Tu Y.-K., Chambrone L. A. (2012). Evidence-based periodontal plastic surgery. II. An individual data meta-analysis for evaluating factors in achieving complete root coverage. *Journal of Periodontology*.

[5] Zucchelli G., Mele M., Stefanini M. (2010). Predetermination of root coverage. *Journal of Periodontology*.

[6] Askan H., Di Gianfilippo R., Ravida A., Tattan M., Majzoub J., Wang H. L. (2019). Incidence and severity of postoperative complications following oral, periodontal, and implant surgeries: a retrospective study. *Journal of Periodontology*.

[7] Franco-Carro B., Barona-Dorado C., Martínez-González M. J. S., Rubio-Alonso L. J., Martínez-González J. M. (2011). Meta-analytic study on the frequency and treatment of oral antral communications. *Medicina Oral, Patología Oral y Cirugía Bucal*.

[8] Borgonovo A. E., Berardinelli F. V., Favale M., Maiorana C. (2012). Surgical options in oroantral fistula treatment. *The Open Dentistry Journal*.

[9] Abrahams J., Berger S. B. (1995). Oral-maxillary sinus fistula (oroantral fistula): clinical features and findings on multiplanar CT. *American Journal of Roentgenology*.

[10] Parvini P., Obreja K., Sader R., Becker J., Schwarz F., Salti L. (2018). Surgical options in oroantral fistula management: a narrative review. *International Journal of Implant Dentistry*.

[11] Hanazawa Y., Itoh K., Mabashi T., Sato K. (1995). Closure of oroantral communications using a pedicled buccal fat pad graft. *Journal of Oral and Maxillofacial Surgery*.

[12] Miller P. D. (1985). A classification of marginal tissue recession. *The International Journal of Periodontics & Restorative Dentistry*.

[13] Lorenzana E. R., Allen E. P. (2000). The single-incision palatal harvest technique: a strategy for esthetics and patient comfort. *The International Journal of Periodontics & Restorative Dentistry*.

[14] Chambrone L., Sukekava F., Araújo M. G., Pustiglioni F. E., Chambrone L. A., Lima L. A. (2010). Root-coverage procedures for the treatment of localized recession-type defects: a Cochrane systematic review. *Journal of Periodontology*.

[15] Guven O. (1998). A clinical study on oroantral fistulae. *Journal of Cranio-Maxillofacial Surgery*.

[16] Zijderveld S. A., van den Bergh J. P. A., Schulten E. A. J. M., ten Bruggenkate C. M. (2008). Anatomical and surgical findings and complications in 100 consecutive maxillary sinus floor elevation procedures. *Journal of Oral and Maxillofacial Surgery*.

